# Differences in prostate tumor characteristics and survival among religious groups in Songkhla, Thailand

**DOI:** 10.1186/s12885-018-5102-2

**Published:** 2018-11-27

**Authors:** Christian S. Alvarez, Eduardo Villamor, Rafael Meza, Laura S. Rozek, Hutcha Sriplung, Alison M. Mondul

**Affiliations:** 1 Department of Epidemiology, University of Michigan School of Public Health, 1415 Washington Heights, Ann Arbor, MI 48103 USA; 2 Department of Environmental Health Science, University of Michigan School of Public Health, 1415 Washington Height, Ann Arbor, MI 48103 USA; 3 Epidemiology Unit, Faculty of Medicine Hat Yai, Prince of Songkla University, Hat Yai District, Songkhla, 90110 Thailand

**Keywords:** Prostatic neoplasms, Survival, Religion, Thailand

## Abstract

**Background:**

The incidence and mortality from prostate cancer is expected to increase in the next decade in Thailand. Despite the perceived lower risk in this population vs. developed, western countries, it is becoming an important public health issue. Prostate cancer incidence varies between the most predominant religious groups in Thailand, Buddhists and Muslims. However limited data is available describing the prostate cancer survival in these two populations. Here we examine differences in prostate tumor characteristics and survival between Buddhists and Muslims in the province of Songkhla, Thailand.

**Methods:**

945 incident prostate cancer cases (1990–2014) from the population-based Songkhla Cancer Registry were used in this analysis. Age, grade, stage, and year at diagnosis were compared across religious groups, using Wilcoxon or Chi-square tests. Kaplan Meier methods were used to estimate the median survival time and 5-year survival probabilities. Cox proportional hazards models were used to estimate hazard ratios (HR) between religious groups and 95% confidence intervals (CI) for mortality in age-adjusted and fully-adjusted models.

**Results:**

Prostate tumor characteristics, age, and year at diagnosis were similar across religious groups. The median survival time after diagnosis of prostate cancer was longer in Buddhists 3.8 years compared with Muslims 3.2 years (*p* = 0.08). The age-adjusted risk of death after prostate cancer diagnosis was higher in Muslims compared with Buddhists (HR: 1.31; 95%CI: 1.00, 1.72). After adjustment by stage and grade, results were slightly attenuated (HR: 1.27, 95%CI: 0.97, 1.67).

**Conclusion:**

Muslims have shorter survival after prostate cancer diagnosis than do Buddhists in Thailand. The reasons underlying this difference require additional investigation in order to design targeted interventions for both populations.

**Electronic supplementary material:**

The online version of this article (10.1186/s12885-018-5102-2) contains supplementary material, which is available to authorized users.

## Background

Worldwide, the overall burden of prostate cancer has increased substantially over the last three decades, with geographical variation in incidence and mortality [1–3]. The highest incidence rates of prostate cancer are observed in Western, developed countries such as the United States (US), (age-standardized incidence rate (ASR): 98.2 prostate cancer cases per 100,000 person-years) [4]. This high incidence can be partially explained by the implementation of population-based screening programs using the prostate-specific antigen (PSA) test in the US population [5]. However, even Western developed countries that do not conduct population-based PSA screening have relatively high incidence rates of prostate cancer (e.g. Canada: 88.9 and the UK: 73.2 prostate cancer cases per 100,000 person-years) [4]. On the other hand, incidence rates of prostate cancer are relatively low in non-Western, less developed regions such as South-East Asia (ASR: 5.5 prostate cancer cases per 100,000 person-years) [4].

Despite these current lower rates in South-East Asia, the burden of disease is expected to increase in this region and other low and middle income countries worldwide [1, 2, 6–8]. In Thailand, prostate cancer is the fourth most common diagnosed cancer and the fourth leading cause of cancer death among Thai men [4]. In southern Thailand, incidence and mortality rates of prostate cancer have increased significantly from 1990 to 2013 at an estimated annual percent change of 4.8 and 5.3% respectively [9]. In addition, prostate cancer rates are projected to continue increasing through 2030, doubling the rates observed in 2013 [9].

Unlike the lower incidence rates in Southeast Asia, prostate cancer mortality rates are relatively high (ASMR: 6.7 deaths per 100,000 person-years) [4]; the mortality-to-incidence ratio (MIR) in Thailand is 0.51, compared to more developed countries such as the US (MIR: 0.09) [9, 10]. The lower survival rates of prostate cancer in many Asian countries is consistent with the large proportion of prostate cancer diagnosed at advanced stages, mostly due by the lack of PSA screening [11, 12]. However, we cannot rule out other factors, such as genetics, access to care, and sociocultural characteristics of Asian populations that may influence disparities in prostate cancer outcomes not only between- but also within- countries [13].

Songkhla is a province in southern Thailand, located on the eastern side of the Malay Peninsula [14]. It has 16 districts with a population of 1.5 million inhabitants [15]. The composition of the population in Songkhla is unique because of the diversity in ethnic/religious groups [14]. Approximately, 25% of the people are Muslims and 75% Buddhists. There are documented health disparities between Buddhists and Muslims in Songkhla, Thailand. These differences are thought to be due, in part, to variability in lifestyle factors because of cultural differences between these groups [16, 17]; for example, studies have reported differences in risk of cancer at several sites, including prostate cancer, as well as differences in risk of other chronic diseases and risk factors, such as metabolic syndrome, cardiovascular diseases and diabetes [17–19]. Prostate cancer incidence rates in Muslims are lower compared to Buddhists (ASR: 8.7 prostate cancer cases per 100,000 person-years in Buddhists vs < 5 in Muslims) [17]. However, to our knowledge, no studies have examined if these differences extend to differential cancer survival between these two religious groups. Therefore, the purpose of our study was to compare the prostate tumor characteristics and the survival time after diagnosis with prostate cancer between Buddhist and Muslim men in Songkhla, Thailand.

## Methods

### Study population

We extracted incident prostate cancer cases from the Songkhla Cancer Registry (SCR) from 1989 to 2014. A detailed description of this registry has been provided elsewhere [20, 21]. Briefly, the SCR is a population-based cancer registry that has actively collected cancer cases in the Songkhla province since 1989. It captures cancer cases from 23 data sources, including governmental and private hospitals as well as the population registration office [20, 21]. The SCR also collects information on age and year at diagnosis, religion, stage, grade as well as date of last contact, date of death, and vital status. The completeness of case ascertainment is greater than 95%, evaluated by capture-recapture methods [22]. This registry delivers high quality data and has contributed data to the International Agency for Research on Cancer (IARC), Cancer Incidence in Five Continent publications since volume VIII [23].

### Data extraction and variables

The 10th revision of the International Classification of Disease (ICD-10) code for malignant neoplasm of the prostate (C61) was used for the extraction of prostate cancer cases. We restricted our analysis to prostate cancer cases diagnosed after 1989, because we assumed that data was incomplete during the first year of registration. In total, 945 prostate cancer cases were diagnosed between January 1, 1990 and December 31, 2014. We further excluded four prostate cancer cases because of missing information on religion.

Religious group (Buddhist or Muslim) is routinely collected in the SCR. Age at diagnosis is recorded as continuous variable (in years). We categorized grade as moderately/poorly differentiated, undifferentiated, or unknown; and stage as localized/regional, distant and unknown. Age at diagnosis was categorized in 5-year groups (e.g. 1990–1994, 1995–1999, 2000–2004, 2005–2009 and 2010–2014). In addition, vital status is recorded as dead or alive. Deaths are ascertained for cancer cases included in the registry through abstraction of vital statistics records, regular scrutiny of hospital records as well as linkage with the referral system and with public and private health care providers [23]. The deaths represent all-cause mortality, not prostate cancer-specific mortality.

### Statistical analysis

Age at diagnosis and prostate tumor characteristics such as grade and stage, as well as year at diagnosis were compared between Buddhists and Muslims. We used the Wilcoxon test to compare median age at diagnosis between the two religious groups, as age was not normally distributed. The chi-squared test was used to compare the distribution of prostate cancer cases by grade, stage and year at diagnosis in Buddhists and Muslims. All tests were considered statistically significant at *p* < 0.05.

The main outcome of interest was survival time, defined as the number of years between date of diagnosis and either date of death or date of last contact. Median survival time as well as 5-year survival probability of prostate cancer were estimated using the Kaplan-Meier method, and differences by religious group were assessed using the log-rank test. Kaplan-Meier survival curves of prostate cancer were obtained for the overall study population and stratified by religious group. To confirm the proportional hazard assumption, we examined Kaplan-Meier plot of survival (S) versus time (T) and log (−log(S)) versus log (T) for Buddhists and Muslims, finding that there was no evidence of violation of the proportional hazard assumption from visual inspection of the survival functions for exposure groups. Further, we included an interaction term between religious group and follow-up time and evaluated its significance using the Wald test; this variable was not statistically significant (*p* = 0.76).

Cox proportional hazard models were used to estimate the hazard ratio (HR) and 95% confidence interval (CI) for mortality. The main exposure considered was religious group as recorded in the registry (Buddhist or Muslim). Models were compared with and without using the following covariates in the model: age, and tumor grade and stage. In addition, we assessed for interaction between religious group and age, grade and stage using product terms. All statistical analyses were conducted in SAS software v 9.4 (SAS Institute, Cary NC).

### Sensitivity analysis

Because of the large number of unstaged and ungraded tumors (78.9 and 46.9%, respectively), we conducted a multiple imputation analysis to impute stage and grade for those missing this information, including age, religion, follow-up time and vital status to predict the missing data. We used the PROC MI statement in SAS to conduct the multiple imputation. We obtained parameter estimates from the multivariable-adjusted Cox proportional hazards regression models for 100 imputed datasets. The parameter estimates were combined for inference using PROC MIANALYZE statement in SAS. We assumed that data were missing completely at random.

To evaluate the effect of period pre- and post-introduction of the universal health coverage by the Thai National Health Office in the early 2000s we examined the religious group-specific median survival time, 12-months, 2- and 5-year survival probabilities, partitioning follow up time as follows: 1990–1999, 2000–2004, 2005–2009 and 2010–2014. Finally, to more tightly control for age and calendar year, we conducted sensitivity analyses using age and calendar year as the time scale in our models.

## Results

Of the 945 prostate cancer cases, 89.2% were Buddhists and the rest Muslims, with a median age at diagnosis of 74 (Interquartile range (IQR) = 67, 79) and 72 (IQR = 68, 77) respectively (Table 1). Of tumors with known grade at diagnosis the majority were moderately/poorly differentiated. Similarly, among tumors with known stage at diagnosis, the majority were distant. In addition, Muslims seem to have a slightly higher proportion of undifferentiated and distant tumors compared to Buddhists. On the other hand, the proportion of ungraded and unstaged tumors is slightly higher in Buddhists compared to Muslims. Furthermore, more than 80% of the cases have been diagnosed since the year 2000 when universal health coverage was introduced in Thailand. We observed no statistically significant differences by religious group for any of the variables examined (Table 1). Age at diagnosis did not differ across categories of stage, grade and year of diagnosis (*p*-values: 0.71, 0.08 and 0.57, respectively), and, as expected, higher grade and stage tumors were statistically significantly more likely to die (data not shown).

**Table 1:** Demographic and prostate tumor characteristics among religious groups in Songkhla, Thailand

Characteristic	Total *N* = 945 n (%) or Median (Q1-Q3)	Religion^a^ (*n* = 941)		
		Buddhists *n* = 843 n (%) or Median (Q1-Q3)	Muslims n = 98 n (%) or Median (Q1-Q3)	p-value
Age	73 (67,79)	74 (67,79)	72 (68, 77)	0.38
Grade				
Well differentiated	186 (19.7)	163 (19.3)	23 (23.5)	0.65
Moderately/Poorly differentiated	247 (26.1)	221 (26.2)	26 (26.5)	
Undifferentiated	69 (7.3)	60 (7.1)	9 (9.2)	
Unknown	443 (46.9)	399 (47.3)	40 (40.8)	
Stage				
Localized/Regional	50 (5.3)	42 (5.0)	8 (8.2)	0.35
Distant	149 (15.8)	132 (15.7)	17 (17.4)	
Unknown	746 (78.9)	669 (79.4)	73 (74.5)	
Year of diagnosis				
1990–1999	177 (18.7)	155 (18.4)	21 (21.4)	0.75
2000–2004	147 (15.6)	133 (15.8)	14 (14.3)	
2005–2009	264 (27.9)	237 (28.1)	25 (25.5)	
2010–2014	357 (37.8)	318 (37.7)	38 (38.8)	

^a^There were 4 missing values for the religious group variable (n = 941)

**Fig. 1:**
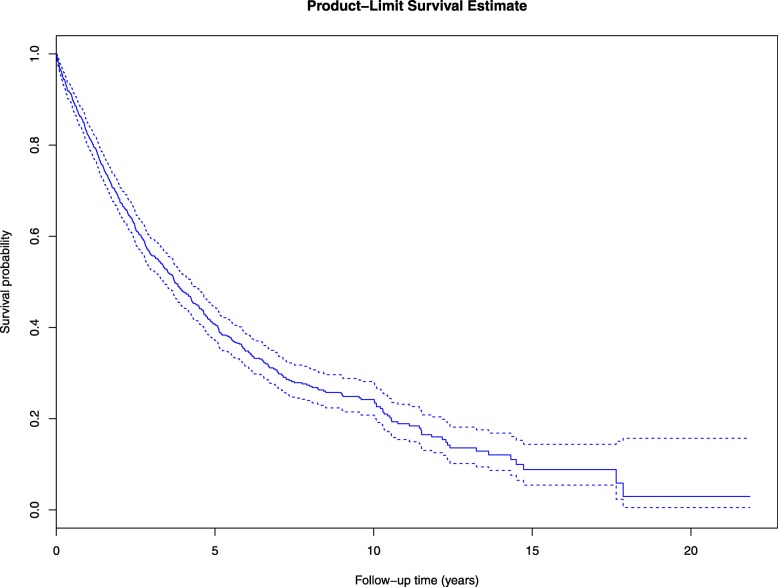
Kaplan-Meier survival curve of prostate cancer in Songkhla, Thailand. Footnote: Overall median survival time: 3.7 (95%CI: 3.4, 4.2). Overall probability surviving after 5 years: 40.6% (95%CI: 37.0, 44.2)

**Fig. 2:**
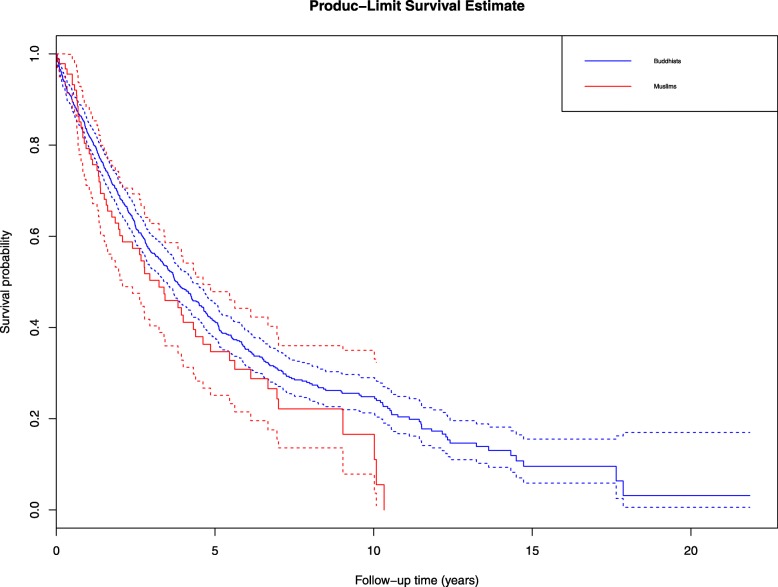
Kaplan Meier survival curves of prostate cancer by religious group in Songkhla, Thailand. Footnote: Log-rank *p* value: 0.08

The overall median survival time after diagnosis of prostate cancer was 3.7 years (95%CI: 3.4, 4.2), and the overall 5-year survival probability was 40.6% (95%CI: 37.0, 44.2) (Fig. 1). Despite the small number of Muslim prostate cancer cases (*n* = 98), we found a borderline significant difference in prostate cancer survival between Buddhists and Muslims (log-rank test, *p* = 0.08). The median survival time was longer in Buddhists 3.8 years (95%CI: 3.4, 4.3) compared to Muslims 3.2 years (95%CI: 2.0, 4.4) (Fig. 2, and Table 2). Similarly, Buddhists have a higher 5-year survival probability of prostate cancer than Muslims, 41.3% (95%CI: 37.4, 45.0) vs 34.7% (95%CI: 23.8, 45.8), respectively (Table 2).

**Table 2:** Median survival time (years), survival probabilities and hazard ratios for death of prostate cancer by religious group in Songkhla, Thailand

Religious group	Deaths	Person-years	Median survival time (y) (95%CI)	5-year survival probability (95%CI)	Model 1	Model 2
					HR (95%CI)	HR (95%CI)
Buddhists	520	3020.4	3.8 (3.4, 4.3)	41.3% (37.4, 45.0)	1.00 (ref)	1.00 (ref)
Muslims	62	279.4	3.2 (2.0, 4.4)	34.7% (23.8, 45.8)	1.31 (1.00, 1.72)	1.27 (0.97, 1.67)

Model 1: Adjusted by age

Model 2: Adjusted by age, grade and stage

We next estimated differences in survival after diagnosis between religious groups. After adjustment for age at diagnosis, Muslim men were more likely to die of any cause post-diagnosis with prostate cancer compared to Buddhist men (HR: 1.31, 95%CI: 1.00, 1.72; *p* = 0.04). This finding was only slightly attenuated after further adjustment for stage and grade at diagnosis (HR: 1.27, 95%CI: 0.97, 1.67; *p* = 0.06). There was no evidence of statistically significant interactions between religious group and age (*p* = 0.64), grade (*p* = 0.22) or stage at diagnosis (*p* = 0.29). In addition, our multiple imputation analysis from 100 imputed datasets yielded similar results for the multivariable-adjusted Cox regression model, the estimated HR for death in Muslims vs Buddhists was 1.28 (95%CI: 0.97, 1.66). Furthermore, the overall stage distribution and by religious groups remain similar after multiple imputation (Additional file 1: Table S1).

Estimates from the overall median survival time (years) by period after partitioning follow-up time show that although overall, there are modest increases in the median survival time and 12-months, 2- and 5-years survival probabilities pre- and post- introduction of universal health access, these increases appear limited to Buddhist men. The 1-year survival probability increases from 77.9% in 1990–1999 to 83.6% in 2010–2014 in Buddhists. On the other hand, among Muslims the 1-year survival probability remained unchanged during 1990–1999: 75.0% and 2010–2014: 75.5% (Table 3). Finally, the three methods used to account for time (person-years, calendar time, and age) yielded similar results in both the age-adjusted and fully-adjusted models (Table 4).

**Table 3:** Median survival time (years), and survival probabilities by period after partitioning follow up time by religious group

Period	Buddhists				Muslims			
	Median survival time (y)	12-month survival prob.	2-year survival prob.	5-year survival prob.	Median survival time (y)	12-month survival prob.	2-year survival prob.	5-year survival prob.
1990–1999	3.0	77.9%	61.0%	30.5%	6.1	75.0%	^a^	^a^
2000–2004	4.3	86.2%	71.6%	43.2%	3.1	74.7%	56.1%	30.0%
2005–2009	3.8	78.9%	65.4%	42.2%	3.4	76.3%	61.8%	21.8%
2010–2014	4.4	83.6%	71.6%	45.1%	2.6	75.5%	58.2%	^a^

^a^Unable to calculate

**Table 4:** Hazard ratios for death of prostate cancer using 3 different methods to account for time: person-years, calendar period and age

Religious group	Model 1:			Model 2:		
	Person-years	Calendar period	Age	Person-years	Calendar period	Age
	HR (95%CI)	HR (95% CI)	HR (95% CI)	HR (95% CI)	HR (95% CI)	HR (95% CI)
Buddhists	1.00 (ref)	1.00 (ref)	1.00 (ref)	1.00 (ref)	1.00 (ref)	1.00 (ref)
Muslims	1.31 (1.00, 1.72)	1.27 (0.97, 1.66)	1.31 (1.00, 1.72)	1.27 (0.97, 1.67)	1.24 (0.94, 1.63)	1.25 (0.95, 1.64)

Model 1: Adjusted by age

Model 2: Adjusted by age, stage and grade

## Discussion

We compared prostate cancer characteristics and survival after diagnosis between Muslim and Buddhist men in Songkhla, Thailand. We found that Muslim men had a higher risk of death after diagnosis of prostate cancer compared to Buddhists, finding which was not fully explained by differences in tumor characteristics at diagnosis. However, the large number of unstaged and ungraded tumors in both groups does not allow for complete adjustment for these factors even when imputation was used to attempt to assign stage and grade to those with missing information. Differences in the distribution of tumor characteristics among those with missing information by religious groups might still explain the observed survival differences.

Overall, the percent of people surviving five years after diagnosis of prostate cancer is lower in Thailand (40.6%) compared to more developed countries such as the US (98.9%). This is partially explained by the widespread use of PSA screening in the US. In Thailand, PSA is not used for screening purposes, although, it is part of the diagnostic workup in patients with suspected prostate cancer. Other factors such as sociocultural characteristics and genetics may explain the differences in prostate cancer outcomes. Importantly, differences in life expectancy between Thailand and more developed countries such as the US is unlikely to explain the difference in survival as the life expectancy in Thai males is close to the US males (72.0 vs 76.1 years) [15, 24].

Our findings are consistent with those from several published studies that suggest that Muslim populations have poorer cancer survival after diagnosis compared to other ethnic and religious groups [23, 25–29]. In Songkhla, Thailand, lower survival rates for oral, breast and cervical cancer have been observed among Muslims compared to Buddhists [25]. Another study conducted in Asian populations found that breast cancer survival is higher among Indian (54%) and Chinese (49%) women compared to Malay women (45%), which is a predominantly Muslim population [26]. In addition, Malay women are more likely to be diagnosed at advanced stages of breast cancer than other ethnic/religious groups [27]. It should be noted that the Muslims in Songkhla, Thailand are predominantly of Malay descent. Similarly, a study conducted in Northern Israel found that Arab women are more likely to be diagnosed at advanced stage for breast cancer, and with more aggressive disease compared to their Jewish counterparts, likely due to differences in genetic susceptibility as well as socioeconomic factors [28]. In the US, a prostate cancer survival study found that risk of death after diagnosis of the disease is 40% higher among South Asian men compared to Whites [30].

It remains unclear why Muslims may experience poorer cancer outcomes. In Thailand, access to healthcare is relatively consistent across the country, and there are ongoing efforts to integrate Muslims in Songkhla into communities along with Buddhists. The Thai government has established policies for cultural assimilation of minority religious groups (e.g. promotion of Thai language and identity). However, there has been resistance to these policies, and cultural differences do persist. For example, some Muslims in southern Thailand speak Yawi (a Malay dialect) as their first language, creating barriers to communicating with healthcare providers who largely speak Thai [25]. In addition, cultural beliefs could be an important barrier for individuals to seek and/or receive healthcare [31]. This may cause delay in diagnosis and treatment for cancer. For example, one study found that Thai Muslims experienced delays in the time from diagnosis to treatment for oral cancer compared to Buddhists, which the authors concluded was likely due to differences in health attitudes, among Muslims in Thailand [32, 33]. Another study that evaluated knowledge and health belief attitudes for oral cancer among Thai Muslims found that they are more likely to use traditional medicine to prevent and treat oral cancer, even if diagnosis of oral cancer was confirmed in a hospital setting [33].

Several studies have reported that the perspectives of sickness and death among Muslims are different than other religious groups [34]. In some studies about health attitudes and knowledge, Muslims have reported a perception of sickness as a God’s proof of their faithfulness [35]. This belief may lead individuals to delay seeking medical care for their cancer, which may partially explain the poorer survival from prostate cancer among Muslim men in Songkhla. Anecdotal evidence from physicians practicing in Songkhla, Thailand suggests that Muslims may be less likely to accept treatment after a diagnosis with cancer, despite having equal access to high quality care. Supporting this, our findings have shown that survival has not improved among Muslims after the introduction of universal health care. Although, reports from a recent WHO report (CONCORD-3) show that prostate cancer survival in Thailand appear to increase by 10% from 1995 to 2014 [36]. In addition, the risk of death after prostate cancer diagnosis appear to increase in Muslims compared to Buddhists after the introduction of the universal healthcare access, e.g. 1990–1999 HR: 0.96, 95%CI: 0.21, 4.19; 2010–2014 HR: 1.52, 95%CI: 1.00, 2.30 (Additional file 1: Table S2). However, this increase is not statistically significant and the number of deaths were small for the earliest period. One possible concern is that if competing risks from other causes of death differ by religious group, this may bias our findings. However, studies comparing chronic disease risk factors between Muslims and Buddhists in Thailand have noted few differences in risk factor profiles by religion [18, 19] . Thus, competing risks seems unlikely to fully explain our findings. Further research is warranted to identify what factors may play a role in the increased risk of death among Muslim men diagnosed with prostate cancer in Thailand and whether similar disparities in cancer survival exist for other cancer sites or chronic conditions.

### Strengths and limitations

Our findings are based on a high quality population-based cancer registry, which allows us to extrapolate the results to the province of Songkhla and the rest of southern Thailand; in addition the completeness of follow up for this cancer registry is very high (> 95%) [22]. Another strength is that the SCR consistently collects information on religious groups that allows us to conduct this type of analysis and identify patterns of the disease in specific groups.

An important limitation of this study is that deaths are not prostate cancer-specific mortality as SCR only collects information on all-cause mortality. This might bias our results by overestimating the prostate cancer deaths. However, it is likely that most of deaths are due to prostate cancer as they were diagnosed at advanced stages (75.8% stage IV at diagnosis). A study conducted in the US SEER registry demonstrated that including those with unknown or undocumented causes of death as prostate-cancer specific deaths led to very little change in the 5-year survival estimation, particularly for those diagnosed at later stages, suggesting that prostate cancer cases diagnosed at later stages are likely to die from their disease rather than from another cause [37]. We could have used data from death certificates from the Thai Ministry of Health to identify prostate cancer specific deaths. However, the quality of death certificates is poor in Thailand [38]. Another limitation of this study is that the number of undetected cases are unknown due to distant communities that may have poor access to health centers, but the capture rate for prostate cancer in Songkhla has been very high. One more limitation is that complete adjustment for stage and grade was not possible because of the large number of unknowns. To address this limitation we conducted sensitivity analyses where we imputed missing stage and grade. The multiple imputation analysis showed similar results for the risk of death between Buddhists and Muslims. In addition, lack of adjustment for other prognostic factors such as socioeconomic status, smoking, comorbidities, may still lead to residual confounding in our results.

## Conclusions

Muslim men had a higher risk of death after diagnosis of prostate cancer compared to Buddhist men. In contrast with Buddhists, prostate cancer survival has remained constant in Muslims even after the introduction of universal health care access. It is important to understand what risk factors may underlie the poorer survival observed in Muslims to design targeted interventions in both populations.

## Additional file

Additional file 1: **Table S1.** Overall stage distribution and by religious groups comparing observed vs imputed data. **Table S2.** Hazard ratios for death of prostate cancer by religious groups after partitioning follow up time. (DOCX 21 kb)

## Abbreviations

ASR: Age-standardized incidence rate
CI: Confidence intervals
HR: Hazard ratios
IARC: International Agency for Research on Cancer
ICD: International Classification of Diseases
MIR: Mortality-to-incidence ratio
PSA: Prostate-specific antigen
SCR: Songkhla Cancer Registry
US: United States
