# Supplementary material for: Differences in prostate tumor characteristics and survival among religious groups in Songkhla, Thailand
Source: BMC Cancer. 2018 Nov 27;18:1175. doi: 10.1186/s12885-018-5102-2 (PMC6260711; doi:10.1186/s12885-018-5102-2)
Supplement: Supplementary file 1 — Table S1. Overall stage distribution and by religious groups comparing observed vs imputed data. Table S2. Hazard ratios for death of prostate cancer by religious groups after partitioning follow up time. (DOCX 21 kb) [file 12885_2018_5102_MOESM1_ESM.docx]

**Differences in prostate tumor characteristics and survival among religious groups in Songkhla, Thailand**

Christian S. Alvarez, Eduardo Villamor, Rafael Meza, Laura S. Rozek,

Hutcha Sriplung, Alison M. Mondul

**Supplementary Appendix**

**Table S1.** Overall stage distribution and by religious groups comparing observed vs imputed data

**Table S2.** Hazard ratios for death of prostate cancer by religious groups after partitioning follow up time.

**Table S1.** Overall stage distribution and by religious groups comparing observed vs imputed data

|  | **Observed data*** | | | **Imputed data (MI=100)**** | | |
| --- | --- | --- | --- | --- | --- | --- |
| **Stage** | **Total** | **Buddhists** | **Muslims** | **Total** | **Buddhists** | **Muslims** |
| **Localized** | 7 (3.5%) | 7 (4.7%) | 0 (0.0%) | 4158 (4.4%) | 4725 (5.0%) | 0 (0%) |
| **Regional** | 43 (21.6%) | 9 (6.1%) | 8 (32.0%) | 22114 (23.4%) | 21263 (22.5%) | 29012 (30.7%) |
| **Distant** | 149 (74.9%) | 132 (89.2%) | 17 (68.0%) | 68229 (72.2%) | 68513 (72.5%) | 65489 (69.3%) |

*Total missing (unknown stage) = 746

**100 imputed datasets, n=945 in each dataset.

**Table S2.** Hazard ratios for death of prostate cancer by religious groups after partitioning follow up time.

| **Period** | **Deaths** | **Person-years** | **Model 1**  **HR (95% CI)**  **Muslims vs Buddhists** | **Model 2**  **HR (95% CI)**  **Muslims vs Buddhists** |
| --- | --- | --- | --- | --- |
| 1990-1999 | 86 | 382.4 | 1.05  (95%CI: 0.25, 4.51) | 0.96  (95%CI: 0.21, 4.19) |
| 2000-2004 | 81 | 476.6 | 1.29  (95%CI: 0.66, 2.50) | 1.26  (95%CI: 0.64, 2.51) |
| 2005-2009 | 170 | 837.1 | 1.26  (95%CI: 0.77, 2.06) | 1.09  (95%CI: 0.66, 1.82) |
| 2010-2014 | 234 | 1380.49 | 1.55  (95%CI: 1.03, 2.34) | 1.52  (95%CI: 1.00, 2.30) |
